# Understanding of Future Prescribers About Antimicrobial Resistance and Their Preparedness Towards Antimicrobial Stewardship Activities in Pakistan: Findings and Implications

**DOI:** 10.3389/fphar.2022.771083

**Published:** 2022-03-08

**Authors:** Khezar Hayat, Noor Fatima, Muhammad Farooq Umer, Farman Ullah Khan, Faiz Ullah Khan, Zia ul Rehman Najeeb, Muhammad Abuzar Ghaffari, Syed Qasim Raza, Wenchen Liu, Chen Chen, Yu Fang

**Affiliations:** ^1^ Department of Pharmacy Administration and Clinical Pharmacy, School of Pharmacy, Xi’an Jiaotong University, Xi’an, China; ^2^ Centre for Drug Safety and Policy Research, Xi’an Jiaotong University, Xi’an, China; ^3^ Shaanxi Centre for Health Reform and Development Research, Xi’an, China; ^4^ Institute of Pharmaceutical Sciences, University of Veterinary and Animal Sciences, Lahore, Pakistan; ^5^ Department of Pharmacology, Central Park Medical College, Lahore, Pakistan; ^6^ Al-Shifa School of Public Health, Rawalpindi, Pakistan; ^7^ Department of Pharmaceutical Chemistry, Faculty of Pharmacy, Bahauddin Zakariya University, Multan, Pakistan; ^8^ Institute of Biochemistry and Biotechnology, University of Veterinary and Animal Sciences, Lahore, Pakistan

**Keywords:** antimicrobial resistance, antibiotics, antimicrobial stewardship programs, medical students, preparedness, Pakistan

## Abstract

**Background:** Insufficient antimicrobial-related training for physicians during their undergraduate education could have a negative impact on their prescribing. Unlike previous studies, this study not only explored the understanding and perception of Pakistani medical students about antibiotics and resistance, but also their preparedness towards antimicrobial stewardship programs.

**Methods:** An online cross-sectional study was undertaken with final-year medical students using a validated questionnaire from January 2021 to May 2021. Descriptive and inference statistics were applied for data analysis.

**Results:** Of 411 students, only 6.3% had undergone antimicrobial resistance (AMR) training. 16.1% of students believed that antibiotics are effective for viral ailments. More than half of the students agreed that AMR is a major healthcare problem in Pakistan (65.9%). Most students viewed poor infection control practices (66.9%), the use of too many broad-spectrum antibiotics (68.4%) for a longer duration (62.8%) with inadequate doses (67.9%) as the causes of AMR. The student’s preparation was insufficient in interpreting microbiological and pathological results (26.3%), selecting the correct antibiotics (22.1%), and awareness of the antibiotic spectrum (20.9%). The median preparedness score showed significant differences with sex (*p* = 0.049), age (*p* < 0.001), institute type (*p* = 0.014), and family income (*p* = 0.006).

**Conclusion:** Pakistani medical students showed adequate understanding of antibiotics, but lacked preparedness for several components of ASPs, including interpretation of microbiological results and spectrum of antibiotics. More steps need to be taken to prepare medical students for AMR and stewardship initiatives adequately.

## Introduction

Antimicrobial resistance (AMR) inherits a significant potential to affect people’s health globally (Ferri et al., 2017). Resistant infections are responsible for one in every three deaths globally, according to the World Health Organization (WHO, 2021), which attributes the increase to the inappropriate use of antimicrobials, including antibiotics. More than 700,000 people worldwide die each year from resistant infections (O’neill, 2016). In the U.S., which provides one-third of all antibiotic prescriptions globally, resistant infections affect at least two million people each year.

Misuse and overuse of antimicrobials contribute to the spread of resistant strains of microbes (Markogiannakis et al., 2021). Antibiotic overuse is becoming increasingly common as a result of the belief that they are safe and that patient satisfaction is dependent on being prescribed an antibiotic. (Foster et al., 2019). The way doctors give antibiotics, patients’ nonadherence to therapy, unjudicial prescribing, inadequate public awareness, self-medication, pharmaceutical industry’s lack of new antimicrobial-related research due to lower economic incentives and rigorous compliance requirements have an obvious impact on the increase of AMR (Baig et al., 2017; Hutchings et al., 2019; Aslam et al., 2020; Gillani et al., 2021). Both the WHO and the UN have recognized the worldwide scope of AMR with the need for global policies (World Health Organization, 2015; United Nations, 2016). The WHO publication “Global Action Plan on Antimicrobial Resistance” states that “there is an international agreement that AMR is a critical public health concern,” underlining the importance of attaining the WHO Global Action Plan’s five strategic goals (2015).

AMR continues to become more widespread in many parts of the world, including Pakistan, and is largely responsible for the rise in healthcare costs and mortality rates (Abrar et al., 2018). In Pakistan, the AMR rate is amplifying at a rapid pace (Bilal et al., 2021). During the last few years, multidrug-resistant and extensively drug-resistant (XDR) bacterial strains have been reported among hospitalized patients. Enterobacteriaceae have demonstrated significant resistance to quinolones (Qamar et al., 2020). XDR typhoid patients showed resistance to most antimicrobials, including ampicillin, chloramphenicol, trimethoprim-sulfamethoxazole, third-generation cephalosporins and fluoroquinolones (Qamar et al., 2018; Azhar et al., 2019; Qureshi et al., 2020). Additionally, several previous studies have reported the continuously swelling burden of resistant pathogens in Pakistan including carbapenem-resistant extended-spectrum beta-lactamase-producing bacteria and methicillin-resistant *Staphylococcus aureus,* in clinical isolates (Bilal et al., 2021). According to the legislation, antibiotics are not allowed to be sold in Pakistan without a valid prescription from a qualified medical practitioner; nonetheless, studies have reported that drug outlets still dispense antibiotics irrationally in several medial ailments including upper respiratory tract infections owing to business interest, poor training, and inadequate knowledge of staff (NIH, 2017; Malik et al., 2021).

In order to address the resistance problem, a significant and permanent amount of research, policies, regulations, and development is required to ensure the growth of new, powerful antibiotics and rationalize the use of antimicrobial across health facilities, agriculture, and the veterinary sector. Antimicrobial stewardship programs (ASPs) are among the potential approaches used globally to cope with the rising level of AMR (Hayat et al., 2019). ASPs primarily prevent unjudicial use of antimicrobials by educating all major stakeholders of the healthcare system, including physicians, pharmacists, and nurses, regarding rationality checking in antimicrobial prescriptions (Science et al., 2019; Scobie et al., 2019).

Medical students need to learn how to prescribe drugs safely and effectively because once graduating and licensed to practice as medical practitioners, they will indeed be prescribing medication daily with minimal supervision (Van Der Voort et al., 2019; Augie et al., 2021). Strong prescribing skill sets can facilitate rational and judicial prescribing, which improves patient benefits, medication compliance, and limit healthcare-associated costs (Essack et al., 2019). Therefore, it is vital to be aware that future medical students have a complete awareness of the growing issue of AMR as they will act as next-generation antimicrobial providers (Haque et al., 2019). Unfortunately, only a limited part of the curricula of Pakistani medical students emphasize on AMR and information on ASPs is completely absent (PMDC, 2011).

Several studies have shown that poor prescribing amongst doctors could be linked to inadequate training during their undergraduate medical education (Okedo-Alex et al., 2019). For example, in a Chinese study, only one-fourth of the medical students sampled had undergone training specifically relevant to antimicrobials and AMR (Hu et al., 2018). In an Italian study, 20% of study respondents deemed antibiotics effective for viral ailments (Scaioli et al., 2015). Consequently, adequate medical education coupled with sufficient training is vital to prepare medical students for the rational use of antimicrobials and approaches to eradicating AMR. The literature is scarce from Pakistani medical students’ understanding about antibiotics and preparedness for antimicrobial stewardship activities. Therefore, the primary objective of this study was to determine the understanding of medical students about antibiotics, perception towards resistance problem, and preparedness for stewardship activities in Punjab, Pakistan.

## Materials and Methods

### Study Design and Population

This cross-sectional study was conducted among medical students from Punjab province, Pakistan, from January 2021 to May 2021. There are 144 medical colleges in Pakistan, and nearly half are in Punjab province (Sadiq, 2021). Medical students in their final year (5th year) of medical education participated in this study.

### Study Instrument

A questionnaire was developed after a comprehensive, relevant literature review (Gupta et al., 2019; Okedo-Alex et al., 2019; Saleem et al., 2019; Struzycka et al., 2019; Van Der Voort et al., 2019; Efthymiou et al., 2020; Tiong and Chua, 2020). Questionnaire validation, including face and content validity, was ensured from an expert team (2 professors and 2 physicians) and a group of potential subjects. Few modifications were made as per the recommendations of experts.

The questionnaire was divided into seven sections with subdivided components, each based on different assessment criteria. The first section was about the demographic components of the students, including gender, age, family income, the professional field of parents, the type of institute, and AMR training. The second section recorded the understanding of students towards antibiotics with options of “Yes” “No” and “Unsure”. One mark was given to a student for each correct response, and their scoring was categorized as either good (≥6 correct responses) or poor (<6 correct responses). The third section was about potential causes of AMR. The fourth section focused on the student’s perception of the problem of AMR. The third and fourth sections were rated on a Likert scale from “strongly agree” to “strongly disagree”. The fifth section determined resources used by medical students for learning about antimicrobial prescribing and AMR with five options including “Always,” “Often,” “Sometimes” “Seldom” and “Never”. The sixth section measured the preparedness of the medical student for numerous activities related to ASPs. Here again, a 5-point Likert scale was chosen to record the response, including “Very good” “Good” “Average” “Poor” and “Very poor”. The last section was about ways to reduce AMR. In this section, students were asked about different approaches through which AMR could be eradicated with options ranging from “Strongly agree” to “Strongly disagree”.

A pilot study was conducted on twenty final-year medical students through which the reliability of the questionnaire was measured. The overall value of Cronbach’s alpha was 0.891, ensuring an acceptable level of internal consistency.

### Sampling

A convenience sampling approach was opted to recruit medical students. The sample size of this study was measured using an online sample size calculator (Raosoft) with a 50% response rate, 5% margin of error, and 95% confidence interval.

### Data Collection

Data were collected using a questionnaire designed on Google forms distributed through social media applications. The students were able to fill the questionnaire by clicking on the link. A brief introduction and objective of the study coupled with informed consent were provided on the first page.

### Inclusion and Exclusion Criteria

This study included only medical students who were in the final year (fifth) of their education. Non-medical students were excluded from this study.

### Statistical Analysis

Using descriptive statistics, the data were displayed as frequency and percentages. As the data had a nonnormal distribution, the median and interquartile ranges (IQRs) were calculated. The study participants’ median knowledge score, perception score, median AMR causes score, median preparedness score, and median eradication score were measured. On continuous data, Kruskal–Wallis and Mann-Whitney tests were used, which were then compared with demographic variables. The IBM Statistical Package for the Social Sciences (SPSS) version 19 was used to analyze all of the data by setting a *p* < 0.05 was set as statistical significance.

## Results

Of 600 students approached, 411 completed the questionnaire (response rate = 68.5%). Most of the students were female (*n* = 299, 72.7%), aged 23–25 years (*n* = 357, 86.9%) and belonged to the public sector medical institutes (*n* = 279, 67.9%). Only a few students had undergone training related to AMR (*n* = 26, 6.3%). As indicated in Table 1, the profession of parents of most of the students was related to medical (*n* = 275, 66.9%) with a monthly income higher than 50,000 (*n* = 242, 58.9%).

**TABLE 1:** Demographic information of participants (*n* = 411).

Variable	Frequency (n)	Percentage (%)
Gender		
Male	112	27.3
Female	299	72.7
Age (years)		
22 years	13	3.2
23–25 years	357	86.9
>25 years	41	10.0
Family income		
<50,000	242	58.9
>50,000	169	41.1
Type of institute		
Public	279	67.9
Private	132	32.1
Type of parents’ profession		
Medial	275	66.9
Non-medical	136	33.1
AMR training		
Yes	26	6.3
No	385	93.7

### Exploration of Students’ Understanding of Antibiotics

Overall, students’ knowledge of antibiotics fell into the category of good (*n* = 256, 62.3%) with a mean score of 7.68 ± 1.72. The students were well aware of the ineffectiveness of antibiotics against viral infections (*n* = 343, 83.5%) and destruction of the normal microbiota of the body, thus increasing the potential for secondary infections (*n* = 358, 87.1%). Most students correctly said that the resistant bacteria could spread across healthcare facilities (*n* = 331, 80.5%); nonetheless, only a few students were familiar with the fact that skipping antibiotics could enhance the resistance potential (*n* = 116, 28.2%). The awareness of students of antibiotic resistance was higher compared to antimicrobial stewardship programs (*n* = 376, 91.5% vs. *n* = 255, 62.0%) as indicated in Table 2. The median knowledge score demonstrated a significant association with gender (<0.001), age (<0.001), type of institute (<0.001), family income (<0.001), and AMR training (<0.001). Female students (Median = 9.00, IQR = 8–9), students aged 23–25 years (Median = 9.00, IQR = 7–9), and students from public institutes (Median = 9.00, IQR = 7–9) had significantly higher median scores compared to other groups (Table 3).

**TABLE 2:** Knowledge about antibiotics.

Questions	Yes	No	Unsure	Correct rate
Antibiotics are useful in treating viral infections	66 (16.1)	343 (83.5)	2 (0.5)	343 (83.5)
Antibiotics can cause secondary infections by killing normal flora	358 (87.1)	42 (10.2)	11 (2.7)	358 (87.1)
Antibiotics can cause allergic reactions	386 (93.9)	15 (3.6)	10 (2.4)	386 (93.9)
A resistant bacterium cannot spread in healthcare institutions	63 (15.3)	331 (80.5)	17 (4.1)	331 (80.5)
Skipping one or two doses does not contribute to the development of antibiotic resistance	277 (67.4)	116 (28.2)	18 (4.4)	116 (28.2)
Cross-resistance is the condition in which the resistance occurs to a particular antibiotic that often results in resistance to other antibiotics, usually from a similar class	353 (85.9)	42 (10.2)	16 (3.9)	353 (85.9)
Pain and inflammation without any possibility of infection are indications for antimicrobial therapy	84 (20.4)	299 (72.7)	28 (6.8)	299 (72.7)
Have you ever heard of antibiotic resistance?	376 (91.5)	28 (6.8)	7 (1.7)	376 (91.5)
Have you been ever taught antibiotic resistance in your curriculum?	365 (88.8)	37 (9.0)	9 (2.2)	365 (88.8)
Have you ever heard of antibiotic stewardship?	255 (62.0)	137 (33.3)	19 (4.6)	255 (62.0)
Have you been ever taught about antibiotic stewardship in your curriculum?	251 (61.1)	128 (31.1)	32 (7.8)	251 (61.1)

**TABLE 3:** Median scores association with demographics.

Variable	Median knowledge score	*p*-value	Median AMR cause score	*p*-value	Median perception score	*p*-value	Median ASP preparedness score	*p*-value	Median AMR eradication score	*p*-value
Gender										
Male	7.00 (5–7)	<0.001	41.50 (38–45)	0.003	38.00 (35–41)	0.838	40.00 (33–44)	0.049	41.00 (37–43)	<0.001
Female	9.00 (8–9)		40.00 (40–40)		37.00 (37–39)		36.00 (36–38)		45.00 (35–43)	
Age (years)										
22 years	5.00 (3–6)	<0.001	42.00 (40–46)	<0.001	39.00 (35–41)	0.572	44.00 (33–47)	<0.001	43.00 (37–45)	<0.001
23–25 years	9.00 (7–9)		40.00 (40–41)		37.00 (37–39)		36.00 (36–40)		35.00 (35–43)	
>25 years	7.00 (6–7)		42.00 (40–45)		39.00 (36–40)		42.00 (40–45)		41.00 (39–44)	
College type										
Public	9.00 (7–9)	<0.001	40.00 (40–40)	0.173	37.00 (37–37)	<0.001	36.00 (36–36)	0.014	35.00 (35–41)	<0.001
Private	7.00 (6–8)		41.00 (37–45)		39.00 (37–41)		40.00 (33–45)		42.00 (39–45)	
Family income										
<50,000	7.00 (8–9)	<0.001	40.00 (40–40)	0.005	37.00 (37–37)	<0.001	36.00 (36–36)	0.006	35.00 (35–35)	<0.001
>50,000	9.00 (6–8)		41.00 (37–45)		39.00 (37–41)		40.00 (33–45)		42.00 (40–45)	
Parents profession										
Medial	9.00 (7–9)	<0.001	40.00 (40–40)	0.906	37.00 (37–37)	<0.001	36.00 (36–37)	0.182	35.00 (35–39)	<0.001
Non-medical	7.00 (6–8)		41.00 (36–45)		40.00 (37–42)		40.00 (32–45.50)		43.00 (40–46)	
AMR training										
Yes	9.00 (5–7)	<0.001	44.00 (35–45)	0.215	40.00 (37–42)	0.033	43.00 (32–46)	0.281	35.00 (35–41)	0.029
No	6.00 (7–9)		40.00 (40–42)		37.00 (37–39)		36.00 (36–42)		42.00 (39–45)	

### Perception of Students About Antibiotic Use and Antimicrobial Resistance

Most of the students agreed that AMR is not only a global concern (*n* = 297, 72.3%) but also a major healthcare problem of the public in Pakistan (*n* = 271, 65.9%) (Table 4). More than half of the students believed that AMR is a major issue (*n* = 273, 66.4%) and that antimicrobials are overused in hospitals where they are rotated (*n* = 245, 59.6%). Many students said that unjudicial antibiotic use is not only unethical (*n* = 251, 61.1%) but could also lead to significant patient harm (*n* = 249, 60.6%). Students believed that adequate education (*n* = 257, 62.5) and the development of newer antibiotics are integral to curtail the AMR issue (*n* = 271, 65.9%). The median perception score was significantly higher among students of the private institute (*p* < 0.001), family income >50,000 (*p* < 0.001), and had undergone AMR training (*p* = 0.033) (Table 3).

**TABLE 4:** Perception about antibiotic use and antimicrobial resistance.

Question	Strongly agree; strongly disagree. N (%)				
	SA	A	N	D	SD
Antimicrobial resistance is a global issue	297 (72.3)	95 (23.1)	15 (3.6)	3 (0.7)	1 (0.2)
Antimicrobial resistance is a serious problem in Pakistan	116 (28.2)	271 (65.9)	20 (4.9)	3 (0.7)	1 (0.2)
Antimicrobial are overused at the hospitals where I have rotated	88 (21.4)	245 (59.6)	61 (14.8)	14 (3.4)	3 (0.7)
Antimicrobial resistance is a significant problem at the hospitals where I have rotated	61 (14.8)	273 (66.4)	65 (15.8)	10 (2.4)	2 (0.5)
Strong knowledge of antibiotic is important in career	143 (34.8)	241 (58.6)	17 (4.1)	5 (1.2)	5 (1.2)
Inappropriate use of antibiotic is professionally unethical	133 (32.4)	251 (61.1)	18 (4.4)	3 (0.7)	6 (1.5)
Inappropriate use of antibiotic can harm patients	144 (35.0)	249 (60.6)	14 (3.4)	3 (0.7)	1 (0.2)
I would like more education on antibiotic resistance	137 (33.3)	257 (62.5)	8 (1.9)	6 (1.5)	3 (0.7)
New antibiotics will be developed in the future that will counter the problem of “resistance”	87 (21.2)	271 (65.9)	39 (9.5)	12 (2.9)	2 (0.5)

SA, strongly agree; A, agree; N, neutral; D, disagree; SD, strongly disagree.

### Potential Cause of Antimicrobial Resistance and Educational Resources

More than two-quarters of the students agreed that the sale of over-the-counter antibiotics (*n* = 248, 60.3%), poor infection control practices (*n* = 275, 66.9%), the use of too many broad-spectrum antibiotics (*n* = 281, 68.4%) for a longer duration (*n* = 258, 62.8%) with inadequate doses (*n* = 279, 67.9%) could aggravate the risk of AMR. Likewise, most students also felt that patients’ non-compliance with their antibiotic therapy (*n* = 257, 62.5%) and extensive antibiotic usage in livestock might lead to AMR (*n* = 268, 65.2%) (Table 5). Gender (*p* = 0.003), age (*p* < 0.001), and family income (*p* = 0.005) showed a significant association with the median score of AMR cause (Table 3).

**TABLE 5:** Potential causes of antimicrobial resistance.

Question	Strongly agree; strongly disagree. N (%)				
	SA	A	N	D	SD
Too many antibiotic prescriptions	118 (28.7)	266 (64.7)	9 (2.2)	5 (1.2)	13 (3.2)
Too many broad-spectrum antibiotics used	103 (25.1)	281 (68.4)	9 (2.2)	11 (2.7)	7 (1.7)
Too long durations of antibiotic treatment	84 (20.4)	258 (62.8)	31 (7.5)	33 (8.0)	5 (1.2)
Dosing of antibiotics are too low	44 (10.7)	279 (67.9)	37 (9.0)	34 (8.3)	17 (4.1)
Excessive use of antibiotics in livestock	81 (19.7)	268 (65.2)	31 (7.5)	23 (5.6)	8 (1.9)
Not removing the focus of infection (e.g. medical devices or catheters)	70 (17.0)	286 (69.6)	25 (6.1)	21 (5.1)	9 (2.2)
Antibiotic sale without prescription from community pharmacies	130 (31.6)	248 (60.3)	14 (3.4)	7 (1.7)	12 (2.9)
Patient non-compliance with antibiotic treatment	129 (31.4)	257 (62.5)	9 (2.2)	10 (2.4)	6 (1.5)
Poor infection-control practices by healthcare professionals	90 (21.9)	275 (66.9)	24 (5.8)	12 (2.9)	10 (2.4)
Paying too much attention to pharmaceutical representatives/advertising	20 (4.9)	8 (1.9)	26 (6.3)	263 (64.0)	94 (22.9)

SA, strongly agree; A, agree; N, neutral; D, disagree; SD, strongly disagree.

**FIGURE 1:**
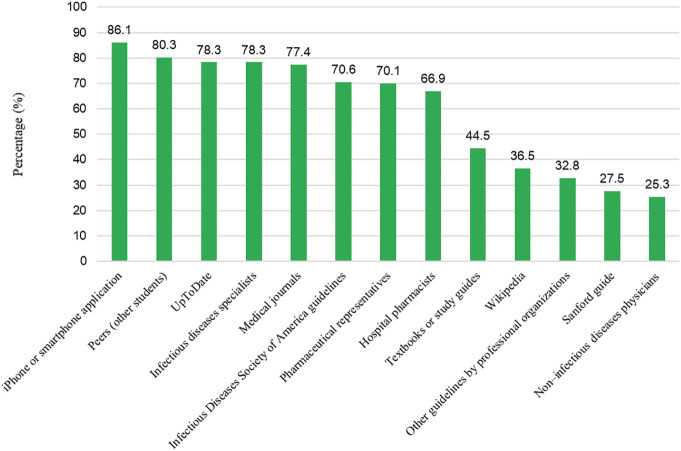
Resources used by medial students for antimicrobial resistant related information (Always and often combined).

Various educational resources utilized by medical students to enhance their antimicrobial related information include iPhone and smartphone applications (86.1%), peers (80.3%), UpToDate (medical app) (78.3%), infectious disease specialists (78.3%), medical journals (77.4%), American guidelines (70.6%), pharmaceutical representatives (70.1%), and hospital pharmacists (66.9%) (Figure 1).

### Preparedness for Antimicrobial Stewardship Activities

The preparedness of students about antimicrobial stewardship activities has been summarized in Table 6. Most of the students rated their preparedness good for accurate infection diagnosis of infection (*n* = 265, 64.5%), when to initiate antibiotic therapy (*n* = 257, 62.5%), de-escalation to narrow-spectrum antibiotics (*n* = 267, 65.0%), switching from oral to IV antibiotics (*n* = 260, 63.3%) and interpretation of antibiograms (*n* = 263, 64.0%). Nevertheless, only a few of the students considered themselves good enough to interpret microbiological and pathology results (*n* = 108, 26.3%), selection of correct antibiotics (*n* = 91, 22.1%), and awareness of the spectrum of antibiotics (*n* = 86, 20.9%). Similarly, students’ preparedness for antibiotic mechanisms was also suboptimal (98, 23.8%). The preparedness towards stewardship activities was significantly higher among students who were female (*p* = 0.049), aged 22 years (*p* < 0.001), belonged to private institutes (*p* = 0.014), and had a family income higher than 50,000 (*p* = 0.006) (Table 3).

**TABLE 6:** Preparedness about antimicrobial stewardship activities.

Question	Very good; very poor. N (%)				
	Very Good	Good	Average	Poor	Very Poor
Making accurate diagnosis of infection	90 (21.9)	265 (64.5)	46 (11.2)	9 (2.2)	90 (21.9)
Interpreting pathology and microbiology results	66 (16.1)	108 (26.3)	220 (53.5)	15 (3.6)	2 (0.5)
Knowing when to start antibiotics	88 (21.4)	257 (62.5)	53 (12.9)	11 (2.7)	2 (0.5)
Choosing the correct antibiotic	79 (19.2)	91 (22.1)	221 (53.8)	18 (4.4)	2 (0.5)
Knowledge of dosing/calculations and duration of antibiotics	76 (18.5)	253 (61.6)	52 (12.7)	23 (5.6)	7 (1.7)
How to de-escalate to narrower spectrum antibiotics	62 (15.1)	267 (65.0)	56 (13.6)	18 (4.4)	8 (1.9)
How and when to transition from intravenous to oral antibiotics	74 (18.0)	260 (63.3)	50 (12.2)	19 (4.6)	8 (1.9)
How to interpret antibiograms	55 (13.4)	263 (64.0)	54 (13.1)	24 (5.8)	15 (3.6)
Understanding spectrums of activity of antibiotics	85 (20.7)	86 (20.9)	213 (51.8)	17 (4.1)	10 (2.4)
Understanding basic mechanisms of resistance of antibiotics	90 (21.9)	98 (23.8)	206 (50.1)	10 (2.4)	7 (1.7)

### Attitude Towards Eradicating Antimicrobial Resistance

Most students agreed that judicial antimicrobials utilization related to formal teaching should be offered to the students (*n* = 279, 67.9%) coupled with the development of institutional treatment guidelines (*n* = 265, 64.5%). Besides, most students said that patients should prohibit the practice of keeping left-over antibiotics (*n* = 266, 64.7%) and self-medication (*n* = 253, 61.6%). Most students remained neutral when they were asked about the “implementation of antimicrobial stewardship programs” (*n* = 202, 49.1%) and “improving diagnostic facilities” (*n* = 186, 45.3%) (Table 7). The median score of eradicating AMR was noted to be significantly associated with gender (*p* < 0.001), age (*p* < 0.001), institute type (*p* < 0.001), family income (*p* < 0.001), AMR training (*p* = 0.029), and parent’s profession (*p* < 0.001) (Table 3).

**TABLE 7:** Attitude towards eradicating antimicrobial resistance.

Question	Strongly agree; strongly disagree. N (%)				
	SA	A	N	D	SD
Educating healthcare professional in terms of appropriate antibiotic prescribing	145 (35.3)	75 (18.2)	189 (46.0)	1 (0.2)	1 (0.2)
Formal teaching on proper usage of antimicrobial agents among health care students	111 (27.0)	279 (67.9)	15 (3.6)	6 (1.5)	0 (0.0)
Implementing antimicrobial stewardships programs	111 (27.0)	91 (22.1)	202 (49.1)	5 (1.2)	2 (0.5)
Rationalizing antimicrobial use in veterinary sector	89 (21.7)	269 (65.5)	45 (10.9)	5 (1.2)	3 (0.7)
Improving diagnostic facilities	128 (31.1)	88 (21.4)	186 (45.3)	5 (1.2)	4 (1.0)
Development of institutional standard treatment guidelines	123 (29.9)	265 (64.5)	13 (3.2)	5 (1.2)	5 (1.2)
Prescribing antibiotics over the phone	35 (8.5)	74 (18.0)	231 (56.2)	38 (9.2)	33 (8.0)
Patient should be advised not to keep part of the antibiotic course for another occasion	94 (22.9)	266 (64.7)	32 (7.8)	9 (2.2)	10 (2.4)
Pharmacists should be discouraged to dispense antibiotics to meet the patients demands	99 (24.1)	92 (22.4)	203 (49.4)	11 (2.7)	6 (1.5)
Self-medication with antibiotics in community should be discouraged	133 (32.4)	253 (61.6)	16 (3.9)	8 (1.9)	1 (0.2)

SA, strongly agree; A, agree; N, neutral; D, disagree; SD, strongly disagree.

## Discussion

This study is the first among Pakistani medical students which assessed their understanding of antimicrobial resistance coupled with its causes and preparedness towards antimicrobial stewardship activities. Our study respondents had a good understanding of certain aspects of antimicrobial use and resistance; nevertheless, their preparedness for certain activities of antimicrobial stewardship programs was suboptimal.

It is widely accepted that antibiotics are unable to cure viral infections, including flu (Alsan et al., 2015). However, 16.1% of our study respondents still believe that antibiotics are effective in viral diseases. A study in Saudi Arabia reported similar findings where 18.1% of medical students perceived that antibiotics could be used against viral infections (Harakeh et al., 2015). Likewise significant gaps in knowledge of medical students about antibiotics including their use in flu and cold have been highlighted (Nogueira-Uzal et al., 2020). Most of the respondents in our study (72.7%) correctly identified that antibiotics should not be administered to relieve pain and inflammation. This agrees with previous studies (Sakeena et al., 2018; Haque et al., 2019). Our respondents better understood the term “antibiotic resistant” compared to “antimicrobial stewardship programs”. This might be because the concept of implementing stewardship programs in hospitals is still new and faces numerous challenges in LMICs, including Pakistan. Previous studies conducted in Pakistan with healthcare professionals and students have also revealed similar findings. The overall understanding of our study respondents regarding antibiotics was still adequate, which might be due to several reasons. First, our respondents were in their last year (5^th^ year) of medical education and had already read about the basic concept of antibiotics and AMR. Second, the parent’s profession of most of our respondents was medical, which could have a positive impact on medical students’ antibiotics-related understanding.

Most of the study respondents believed that AMR is a global threat, and its intensity is also higher in Pakistan. Numerous studies have already revealed that AMR affects nearly every corner of the world and demands certain serious steps to help combat it (Nathan, 2020; Watkins and Bonomo, 2020). In Pakistan, the situation of AMR could be grimmer due to inadequate diagnostic laboratories, the poor performance of the infection control committee, lack of a sufficient number of infectious disease physicians, and antibiotic sales without a prescription (Hayat et al., 2019; Atif et al., 2021). Interestingly, more than half of our survey respondents also agreed that there is an overuse of antibiotics in health facilities where they are getting training. Several studies from Pakistan have also confirmed this point, making the situation severe (Mustafa et al., 2020; Mustafa et al., 2021).

Most of our respondents said that the discovery of newer antibiotics could counter the problem of AMR. The WHO has already stated that global efforts to curtail the global resistance issue have been threatened due to the lack of new antibiotic molecules. To address this issue, the WHO has issued a list of pathogens against which antibiotics should be prepared in priority (Tacconelli et al., 2018).

As indicated in previous studies (Atif et al., 2019; Hayat et al., 2019; Asghar et al., 2020; Malik et al., 2021; Umair et al., 2021), our study respondents believed that the use of antibiotics in livestock, inadequate dosing and duration of antibiotics, antibiotic sale without prescription, and excessive use of broad-spectrum antibiotics are potential causes of AMR. These irrational practices are common in LMICs, including Pakistan. For example, a recent study conducted in Pakistan found antibiotic dispensing without any prescription from more than half of the drug stores (Malik et al., 2021). Similarly, in another Pakistani study, the trend to use antimicrobials in livestock was noted to be very high (Mohsin et al., 2019).

Our study respondents were well prepared regarding certain activities of stewardship programs, including infectious disease diagnosis and initiation of antibiotic therapy coupled with dosing, which is in line with previous studies conducted on South and East African medical students (Wasserman et al., 2017; Lubwama et al., 2021). Nonetheless, students lacked preparedness regarding knowledge of the spectrum of antibiotics and proper antibiotic selection, which are integral components of judicial antibiotic prescribing. Similar results have previously been reported in which more than half of the students agreed that they are unable to know the spectrum of antibiotics and the selection of the correct antibiotics (Wasserman et al., 2017). This might be due to the lack of adequate antimicrobial education of students, which could have a negative impact on the preparedness of students for stewardship programs. The students in our study (67.9%) were also agreed that formal teaching pointed towards antimicrobial agents should be initiated for medical students. We have previously highlighted this point that there is an urgent need to review the curriculum of medical students, including pharmacy students, considering the need for antimicrobial education (Hayat et al., 2021). Unfortunately, developed countries like the U.S. are also facing similar challenges (Abbo et al., 2013).

Most respondents utilized iPhone or smartphone applications, peers, UpToDate, medical journals, and IDSA guidelines. This could have a positive impact on the understanding of antimicrobials and AMR among medical students. Similar findings have been reported previously (Saleem et al., 2019; Hand et al., 2021).

The presence of and adherence to institution-based standard treatment guidelines (STGs) is integral in managing infections as they have proven benefits in reducing mortality, morbidity, and therapy cost (Wiedenmayer et al., 2021). Besides, STGs also minimize the probability of AMR emergence. Our study respondents agreed that STGs should be devised in every institution to help limit the increasing risk of AMR. Unfortunately, most health facilities in Pakistan lack STGs, specifically primary and secondary healthcare facilities, which could contribute to unjudicial antimicrobial use in hospitals (Mustafa et al., 2021). Among other AMR eradication strategies to which our respondents agreed include patient counseling about leftover antibiotics and discouraging self-medication with antibiotics in the community. These practices have already been reported in Pakistan (Gillani et al., 2017; Akram et al., 2019). The government should launch mass-level campaigns to educate the public about rational antibiotic use and AMR risk.

There are certain potential limitations of our study. First, the study was conducted on a limited number of medical students in a single province (Punjab) of Pakistan, and the results are not generalizable to the entire study population. However, Punjab is the largest province in Pakistan, where more than 50% of the population of Pakistan resides, and the medical education in Punjab is exemplary for other provinces. Second, the study was carried out online with a convenience sampling approach that could show bias; nevertheless, it was the only suitable option to collect data due to the ongoing COVID-19 pandemic (Hayat et al., 2020). Third, we were unable to determine cause and effect owing to the typical nature of cross-sectional studies. Despite the above-mentioned limitations, the findings of our study are robust and helpful in designing policies to improve antimicrobial education in medical institutes in Pakistan.

## Conclusion

Our study respondents had an acceptable level of understanding of antibiotics with a positive perception of AMR. Nevertheless, their preparedness towards numerous aspects of antimicrobial stewardship programs was suboptimal. Formal teaching about rational antimicrobial usage, developing institutional treatment guidelines, and limiting self-medication were practical approaches suggested by the respondents to tackle AMR.

### Recommendations

Our study findings suggest that the government should make serious efforts to develop outcome-based curricula for medical education that emphasize AMR education and stewardship. Numerous learning methods, including clinical scenarios, interactive workshops, seminars, conferences, and awareness campaigns, should be opted to equip better medical students about integral components of AMR and stewardship programs. It would be instrumental in providing AMR-related training to medical students in the form of a course that should be part and parcel of their medical curricula. Aside from that, there is a pressing need to adhere to the WHO’s AMR education and training framework, which could assist all medical practitioners in restoring health by conserving the potential of antimicrobials.

## Data Availability

The original contributions presented in the study are included in the article/supplementary material, further inquiries can be directed to the corresponding author.
